# Predatory Functional Morphology in Raptors: Interdigital Variation in Talon Size Is Related to Prey Restraint and Immobilisation Technique

**DOI:** 10.1371/journal.pone.0007999

**Published:** 2009-11-25

**Authors:** Denver W. Fowler, Elizabeth A. Freedman, John B. Scannella

**Affiliations:** Museum of the Rockies, Montana State University, Bozeman, Montana, United States of America; University of Oxford, United Kingdom

## Abstract

Despite the ubiquity of raptors in terrestrial ecosystems, many aspects of their predatory behaviour remain poorly understood. Surprisingly little is known about the morphology of raptor talons and how they are employed during feeding behaviour. Talon size variation among digits can be used to distinguish families of raptors and is related to different techniques of prey restraint and immobilisation. The hypertrophied talons on digits (D) I and II in Accipitridae have evolved primarily to restrain large struggling prey while they are immobilised by dismemberment. Falconidae have only modest talons on each digit and only slightly enlarged D-I and II. For immobilisation, Falconini rely more strongly on strike impact and breaking the necks of their prey, having evolved a ‘tooth’ on the beak to aid in doing so. Pandionidae have enlarged, highly recurved talons on each digit, an adaptation for piscivory, convergently seen to a lesser extent in fishing eagles. Strigiformes bear enlarged talons with comparatively low curvature on each digit, part of a suite of adaptations to increase constriction efficiency by maximising grip strength, indicative of specialisation on small prey. Restraint and immobilisation strategy change as prey increase in size. Small prey are restrained by containment within the foot and immobilised by constriction and beak attacks. Large prey are restrained by pinning under the bodyweight of the raptor, maintaining grip with the talons, and immobilised by dismemberment (Accipitridae), or severing the spinal cord (Falconini). Within all raptors, physical attributes of the feet trade off against each other to attain great strength, but it is the variable means by which this is achieved that distinguishes them ecologically. Our findings show that interdigital talon morphology varies consistently among raptor families, and that this is directly correlative with variation in their typical prey capture and restraint strategy.

## Introduction

Birds of prey or “raptors” (Accipitridae: hawks, kites, and eagles; Falconidae; Pandionidae: the osprey; and Strigiformes: owls) are among the most familiar and geographically widespread of all vertebrates. They are admired for their predatory ability, but surprisingly little is known about the patterns of physical interaction between predator and prey during capture and dispatch.

It has often been assumed that raptors mainly use their sharp talons (a specific term referring only to the claws of birds of prey) to kill their prey [1]. This misconception is rooted in the difficulty of tracking and observing aerial predators after a prey item has been captured [2]–[5]. Even with modern technology, observation of post-capture predator-prey interaction in the wild is still largely opportunistic; consequently, prey immobilisation behaviour is greatly understudied. The term “immobilisation” (where the victim is no longer capable of movement or retaliation) is preferred to “killing” because in some accipitrids at least, if the prey is suitably immobilised and subdued, the raptor will commence feeding even before the death of its victim [6]–[8].

From the limited number of published reports, it is apparent that a combination of the initial strike impact, constriction by the feet, attacks from the predator's beak, dismemberment, and piercing of vital organs by talons are variably employed by raptors to immobilise prey [1], [6], [9], [10]. Experiments in which caged wild raptors were offered live laboratory mice as prey [6], [10]–[14] found that if the initial strike does not kill a prey item outright, the long, recurved talons are not then used to deliver the killing blow. Rather, the mouse is precisely grasped with one or both feet, targeting the victim's head and torso to avoid retaliatory bites and kicks [6]. The raptor's elongate digits are wrapped around the victim; the talons help to restrain the animal and prevent escape. The toes squeeze strongly (either constantly: Falconini; or intermittently: Accipitridae), causing thoracic compression and death by asphyxiation [1], [10]. Squeezing may force talons into the flesh, piercing internal organs and hastening death (especially in *Accipiter* [15]), although this is not commonly observed [10]. During asphyxiation, occasional blows to the head are delivered to attempt to damage the central nervous system, or to the neck (in the case of Falconini) in order to break it. Falconini might even attempt beak attacks mid-air, if the prey item is held onto in flight [9].

Recent studies of raptor predatory functional morphology have included skull morphometrics [8], pes tendon systems [16], musculoskeletal mechanics [17], and the hindlimb as a whole [18], [19] Claw morphology has received virtually no attention at all, which is surprising given its importance to predatory success [6]. Indeed, little literature considers claw morphology for any birds. In the most detailed study, Einoder and Richardson [20] took foot measurements (including claws) from a range of extant Australasian raptors, looking for ecological links with prey choice, size, “hunting-killing technique”, and phylogeny. Csermeley and Rossi [21] investigated whether the D-I and III claws of raptors could be differentiated from non-raptors. The few other studies on claws were primarily concerned with ecological analogues of non-raptor fossil birds, usually only taking measurements for D-III [22]–[25]. Most of these previous analyses made the *a priori* assumption that their choice of measured claw or claws was the most important, which may not be true for all taxa (indeed, our study shows that it is not true; see Supporting Information Text S1). Consequently, the authors were not able to note any patterns of interdigital claw size distribution occurring within or among taxa. Studies in which all claws were considered either only measured claw curvature [22] or used only toe-to-claw length ratios (not considering curvature) [20], and so were unable to note many of the patterns we describe here, or their possible functional correlates.

**Figure 1:**
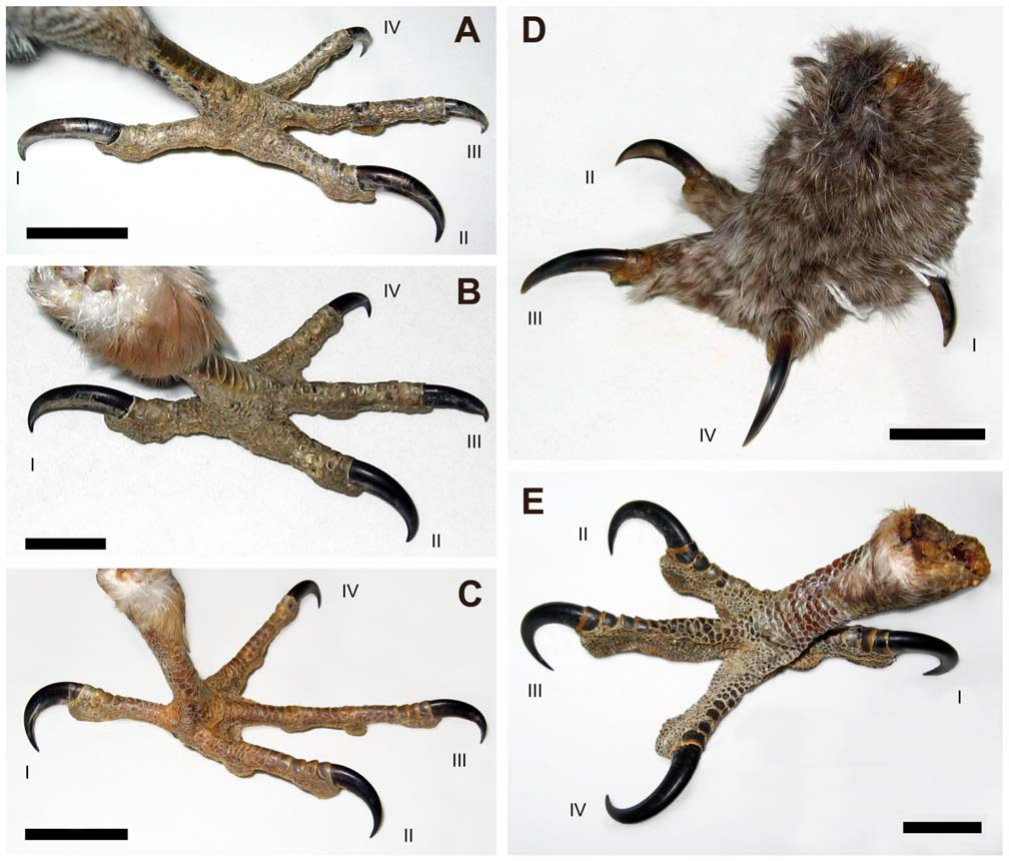
Feet of representative raptors. Note the digit length and relative enlargement and curvature of claws within each foot: Accipitridae bear hypertrophied talons on D-I and II; Falconidae have only modest talons on each digit and only slightly enlarged D-I and II; Strigiformes bear large talons with comparatively low curvature on each digit; Pandionidae have enlarged, highly recurved talons on each digit. (A) Accipitridae: goshawk, *Accipiter gentilis*, MOR OST-1276; (B), Accipitridae: red-tailed hawk, *Buteo jamaicensis* MOR OST-1275; (C) Falconidae: peregrine falcon, *Falco peregrinus*, MOR OST-1265; (D) Strigiformes: great grey owl, *Strix nebulosa*, MOR OST-1284; (E) Pandionidae: osprey, *Pandion haliaetus*, MOR OST-1268.

In an initial survey of specimens, we found that claw size distributions vary conspicuously and consistently among families of raptors (Figure 1), an observation which has gone largely unnoticed in previous studies (although see Einoder and Richardson, 2007 [20]. Indeed, in many illustrated guides [26] claw morphology and relative size is often incorrectly illustrated, being overlooked in favour of plumage. To investigate further, we necessarily took a more complete approach than in previous analyses, measuring each claw of each digit, and also lengths of the toes and tarsometatarsus. These data were then assessed alongside new and published observations of raptor predatory activity to look for consistent patterns of behaviour that correlated with variation in talon and foot morphology at the family level. Our method is preferable to those of previous workers as it encompasses a full range of measurements, treating the foot as a whole, and because previously published qualitative accounts of predatory behaviour did not consider the influence of variation in talon morphology, necessitating reinterpretation which we present here.

## Materials and Methods

Previous authors [e.g.], [19, 21] have considered Falconidae and Accipitridae together as Falconiformes (or equivalent), but recent molecular phylogenetic analyses suggest that this single grouping is paraphyletic [27], [28; although the morphological study of Livezey and Zusi retains this relationship], [29], [30]. Regardless of whether or not this is the case, distinct variation in predatory morphology between the two families renders their treatment as a single group inappropriate for this study; thus, they are referred to separately throughout this paper. Where appropriate, we follow the higher level molecular phylogeny for birds presented by Hackett et al [28].

Recently published molecular phylogenies for Falconidae [31] and Accipitridae [32] necessarily presented new definitions of raptor taxonomic nomenclature (since some traditionally recognized subfamilies of Accipitridae were found to be paraphyletic). We follow the new taxonomic nomenclature of Griffiths et al [31], [32] for Falconidae (Herpetotherinae and Falconinae = Caracarini + Falconini) and Accipitridae (Accipitrinae = Elanini, Gypaetini: Gypaetina + Pernina, Accipitrini: Harpiita + Aquita + Accipitrita, including subclades of Accipitrita: Buteonines (1), Buteonines (2), Sea Eagles, and ‘Accipitrines and *Circus*’) with the exception that we retain the genera *Pandion* and *Sagittarius* within their own monospecific families (Pandionidae and Sagittariidae, respectively) rather than as the basalmost members of the Accipitridae. This slightly less inclusive usage facilitates greater clarity when discussing family-level trends in relative claw size, and retains a monophyletic Accipitridae. A list of observed taxa arranged in a phylogenetic context can be found in Supporting Information Table S3.

As a consequence of lack of previous studies, it has never been demonstrated that raptor talon morphology varies as a result of either gender or ontogeny. Initial observations of specimens showed unequivocally that gross claw morphology does not vary due to these factors, and that general family-level trends are consistent regardless of gender, ontogenetic stage (only post-hatchlings were observed), whether the foot measured is a right or left, and overall body size of the individual bird or species.

A total of 1244 specimens (223 raptors and 1021 non-raptors) and 223 photographs (177 raptor, 46 non-raptor) were studied with regards to claw size proportions. A subset of precisely measured specimens was analysed for quantitative assessment of the observed trends.

In order to take precise measurements of all four digits it is essential to have feet preserved with each of the toes splayed apart, with good lateral views of each claw for photographing. This precludes most preserved skins and mounts for measurement purposes. We surveyed hundreds of preserved skins and mounts held at the Dept. of Ecology (Montana State university, Bozeman, MT), and selected for measurement all raptor specimens where each talon could be photographed adequately for accurate measurement. We also included 26 isolated feet with splayed toes, held at the Museum of the Rockies, (Montana State University, Bozeman, MT), and 4 additional specimens of exotic species were sampled from collections held at the American Museum of Natural History, New York. In total, we measured 34 feet, from 24 species of raptor. We also measured 10 non-raptor taxa in order to represent claw size distributions amongst non-raptors.

Specimens that could not be photographed adequately for precise measurement were used to assess the validity and consistency of size distribution trends inferred from measured specimens. We observed 775 skins (113 raptor & 662 non-raptor), 409 mounts (65 raptor 344 non-raptor), 15 skeletons (11 raptor & 4 non-raptor), and 223 photographs where relative claw sizes were clearly visible (177 raptor, 46 non-raptor).

**Figure 2:**
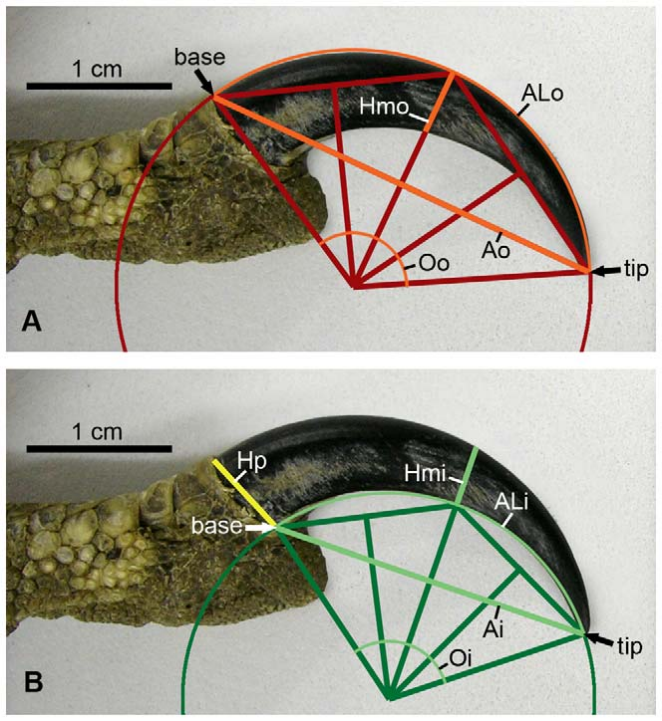
Size and curvature measurements taken from each claw, using methodology of Pike and Maitland (2004). (A) Outer curvature measurements. ALo, arc length from claw base to tip; Ao, straight line (chord) distance from claw base to tip; Hmo, height of claw at midpoint; Oo, angle of curvature. (B) Inner curvature measurements. ALi, arc length from claw base to tip; Ai, straight line (chord) distance from claw base to tip; Hmi, height of claw at midpoint; Hp, height of claw at base; Oi, angle of curvature.

A variety of measurements was taken for each claw of every digit on each foot sampled (following the method of Pike and Maitland [24], Figure 2). Length and angle measurements were taken on close-up photographs of the claws using the measure tool in Adobe® Photoshop®. Additional data (digit length, tarsometatarsus length, gender, maturity, body weight at death) were recorded when possible. In the 4 specimens where talons lacked their keratin sheaths, the bony core alone was measured, with a reconstructed tip if broken. Although this reduced linear measurements and curvature compared to sheathed claws, relative size and curvature among digits should not be affected (this was confirmed by measurement of claws with removable sheaths). In total over 1500 individual measurements were taken (see Supporting Information Table S1).

Claw curvature radii were calculated for both inner and outer curvature from the angle of curvature and the length of the chord created by the line drawn from claw base to tip. Previous workers have used either outer or inner curvature for their analyses, but we took both sets of measurements in case one later proved more informative than the other. The radius and angle of claw curvature were subsequently used to calculate claw “size”: the arc length (ALo for outer measurements, Ali for inner measurements, Figure 2, see Supporting Information Text S1 for calculation formulae) of the claw. Comparison of AL between taxa was assessed by either comparison relative to other talons (ie. which digit bears the largest talon, and by what magnitude), or relative to the toe length of the foot. To remove the effect of body size, measurements were standardised to ratios relative to the talon size of D-III and IV (see Supporting Information Table S1). The D-IV ratio was used for most comparisons, because it is the smallest claw in nearly all taxa measured, and has less variation in relative size among taxa.

We reinterpreted previously published qualitative accounts of predatory behaviour based on insight gained from our analysis of talon morphology. These are complemented by behaviour data taken from our observation of over 170 video sequences showing raptors and prey during capture, immobilisation, and ingestion (Supporting Information Table S2). Behaviour data are much more widely available for North American and European taxa, so our inferences for family-level predatory behaviour should be treated as tentative for taxa from other geographic areas.

Family-level trends in relative toe and claw dimensions that were noted during visual examination were confirmed with two-sample t-tests and paired t-tests, assuming equal or unequal variances where appropriate. T-tests were used rather than ANOVAs so that we could statistically test the exact combination of measurements and taxa that evoked a visually observed trend, instead of testing all characters at once and having to filter the important trends from minor variations.

**Figure 3:**
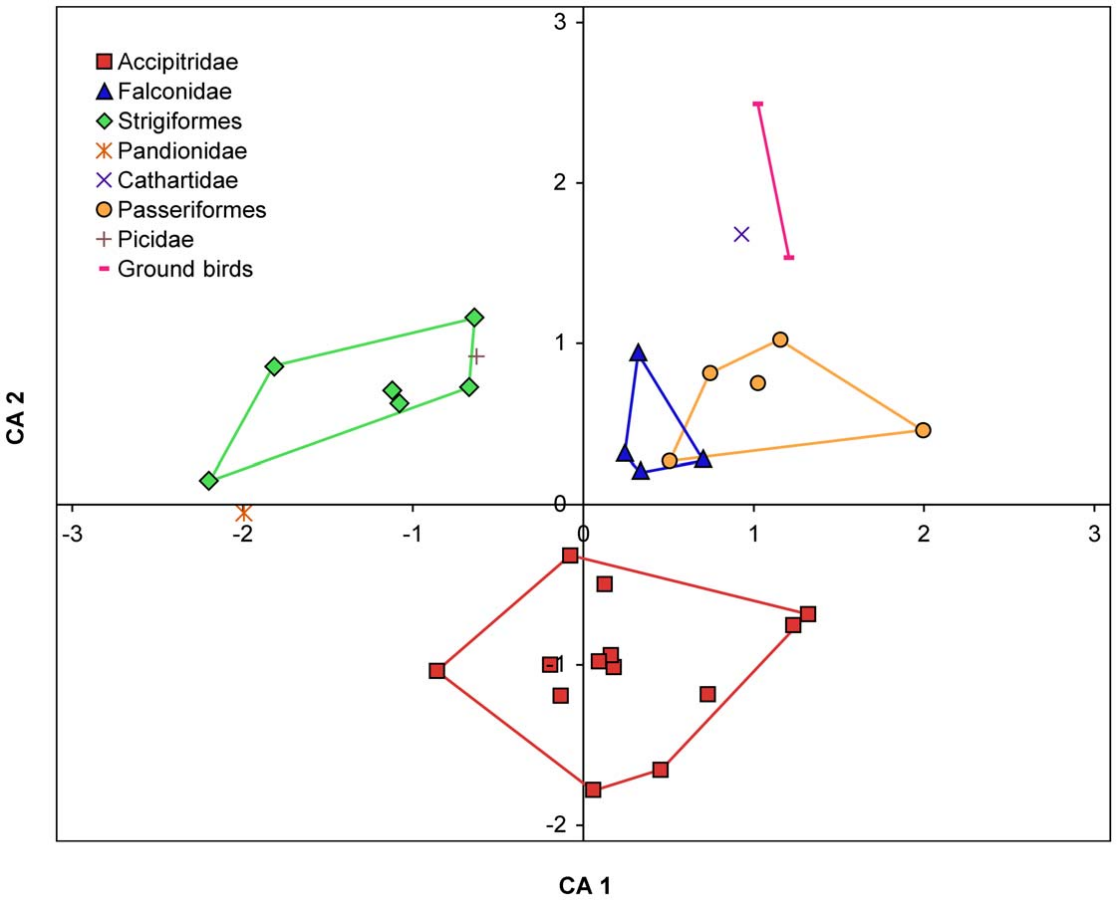
Correspondence analysis of relative claw and toe sizes on each digit amongst taxa. Raptor taxa group tightly into discrete family clusters. Axis 1 accounts for 58.1% of the variation within the data set, and Axis 2 accounts for 25.5%. Axis 1 is controlled by the sizes of all claws relative to toe 3. Axis 2 is mainly driven by the sizes of claws 1 and 2 relative to claw 4 and toe 3, with relative toe sizes also influencing taxa distribution. Measurement ratios used: claw-I/claw-IV, claw-II/claw-IV, claw-III/claw-IV, claw-I/toe-III, claw-II/toe-III, claw-III/toe-III, claw-IV/toe-III, toe-I/toe-IV, toe-II/toe-IV, toe-III/toe-IV. These ratios are displayed because they best explained the variation within the data set using the fewest number of axes.

Correspondence analyses were run using the R language and environment for statistical computing (version 2.7.1 for Mac OSX: www.R-project.org; [33]) to determine whether relative claw sizes can be used to separate specimens into discrete family-level clusters. Correspondence analysis was used rather than principal components analysis because correspondence analysis is better for ecological data, being less susceptible to the distorting effects of outliers and nonlinear distribution of data points. To eliminate the effects of bodysize, raw measurements of claw dimensions were converted into ratios. Various combinations of relative size and curvature measurements were input into a total of 14 correspondence analyses, beginning with the full data set and then testing subsets to remove measurement ratios that produced noise, until we found the fewest number of ratios needed to produce clear clustering. Optimal clustering occurred when outlines of family groups had little or no overlap with each other. Analyses with the tightest clustering also had the highest eigenvalues for the first three axes. The final result, Figure 3, yields similar clusters to our earlier analyses but with tighter clustering and fewer outliers. Detailed explanations of the vetting process and removal of specimens and measurements from the final correspondence analysis are in the Supporting Information Text S1.

## Results

In most birds, the claw of D-III exhibited the least curvature. Paired tailed t-tests confirm that the claw of D-III is significantly less curved than D-I (*t*_43_ = 6.872, *p* = 0.000), II (*t*_43_ = 6.851, *p* = 0.000), and IV (*t*_43_ = 5.579, *p* = 0.000). The opposite was found in the flicker, *Colaptes auratu*s (Picidae). This is probably because the zygodactyl feet of Picidae are specialized for trunk-climbing such that only D-II and D-III (the most curved claws) project anteriorly; these two claws must grip and gain purchase on the trunk when climbing.

Within non-raptor perching birds, D-I and III bear the largest claws (D-I/D-IV mean = 1.455, σ = 0.398; D-II/D-IV mean = 1.081, σ = 0.086, D-III/D-IV mean = 1.294, σ = 0.089; Table 1), and D-I is, on average, 12.5% larger than D-III. In general, D-IV bears the smallest claw; the order of size therefore being (greatest first): I, III, II, IV. In non-raptor ground birds, the D-I claw varies in size, but the relative sizes of claws among D-II, III, and IV are the same as with perching birds.

**Table 1:** Mean and standard deviation of claw sizes (outer arc lengths) of D-I, II, and III relative to D-IV, and D-II relative to D-III.

		D-I/D-IV		D-II/D-IV		D-III/D-IV		D-II/D-III	
	n	mean	σ	mean	σ	mean	σ	mean	σ
Accipitridae	15	1.803	0.266	1.653	0.154	1.196	0.074	1.387	0.162
Falconidae	4	1.273	0.120	1.127	0.083	1.105	0.012	1.019	0.070
Pandionidae	1	0.951	-	0.899	-	0.922	-	0.975	-
Strigiformes	9	1.045	0.060	1.191	0.044	1.158	0.055	1.030	0.051
Non-raptors	8	1.308	0.439	1.084	0.076	1.286	0.091	0.847	0.082
perching birds	6	1.455	0.398	1.081	0.086	1.294	0.089	0.840	0.094
ground birds	2	0.867	0.192	1.093	0.053	1.263	0.130	0.867	0.047

n = number of individuals, σ = standard deviation. For specimens where both feet were measured, the average of those measurements are used here.

**Figure 4:**
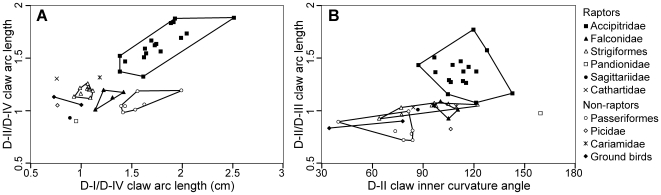
Bivariate plots of claw morphology illustrating family level differentiation. (A) Raptors show variation in the relative size of D-I and II claws among families; Accipitridae have hypertrophied D-I and II claws; Falconidae have only slightly enlarged claws on D-I and II; Strigiformes and Pandionidae have claws that are all similar in size. (B) Falconids can be most easily differentiated from non-raptors by the greater inner curvature of D-II claw, and that the D-II/D-III claw arc length ratio is greater than or very close to 1.

Raptors (other than the osprey) can be distinguished from non-raptors by a D-II claw that is larger than D-III (the opposite is true for non-raptors; Table 1). Within raptors, Accipitridae, Falconidae, Pandionidae, and Strigiformes show consistent talon size distributions at the family level and can be distinguished from each other by this alone (Figure 3, 4; Table 1). Use of other measured variables (claw curvature, tarsometatarsus length) can aid in their identification, and differentiation from non-raptors.

Multiple correspondence analyses were run for the entire dataset, and several vetted subsets that removed potential problematic taxa (see Supporting Information Text S1). Despite the removal of taxa, the results of all analyses were very similar, with each demonstrating distribution of raptor species into separate family clusters. The optimal correspondence analysis included all measured specimens except juveniles and specimens lacking keratin claw sheaths. The tightest clustering and highest eigenvalues resulted from 10 measurement ratios: size of claws I/IV, II/IV, III/IV; length of toes I/IV, II/IV, III/IV; and size of claws I, II, III, and IV relative to length of toe III.

Correspondence analysis of the vetted data set (Figure 3) produced the tightest clustering of raptor species into family groups, and also explained the most amount of variance along the first two axes. The x-axis (which explains 58.12% of the variance) is controlled by the sizes of all claws relative to toe 3. The y-axis (25.50% of total variance) is mainly driven by the sizes of claws 1 and 2 relative to claw 4 and toe 3, with relative toe sizes also influencing taxa distribution. Along the x-axis Strigiformes and Falconidae are clearly separated and cluster tightly amongst themselves. Accipitridae cluster together near the center of the x-axis, yet are the only group on the negative side of the y-axis, suggesting that the morphological characters that are driving variation along the y-axis (size of claws I and II relative to other claws) are highly useful in distinguishing Accipitridae from other bird groups.

### Accipitridae

Accipitrids (Figure 1A, B) are characterised by strikingly hypertrophied talons on D-I and II (Table 1) significantly larger than in all other raptor families (D-I/D-IV *t*_21_ = 8.998, *p*≪0.001; D-II/D-IV *t*_23_ = 10.615, *p*≪0.001). D-III and IV talons are more “normal” in absolute size (although in the bald eagle D-III and IV are larger and more curved than expected: D-III inner curvature *z* = 2.561, *p* = 0.005; D-IV inner curvature *z* = 2.002, *p* = 0.023). There is some evidence to suggest that members of the genus *Accipiter* have more narrow toes than is typical for Accipitridae (consistent with the findings of Einoder and Richardson [20].

### Falconidae

Falconidae (Figure 1C) can be differentiated from other raptors by smaller, subequally sized talons on each digit (Table 1), and an elongate D-III toe excluding the talon. However, their relative talon sizes are comparable to those observed in passerines, making it difficult to distinguish between them based on claw size distribution alone (also noted for D-I and III by Csermeley and Rossi [21]).

We found evidence that Falconidae can be weakly separated from passerines (Figure 3, 4) by a number of factors. Their D-I and II talons relative to the length of the respective toes are significantly larger than in passerines (claw1/toe1 *t*_7_ = 3.175, *p* = 0.008, claw2/toe2 *t*_7_ = 3.808, *p* = 0.003). In Falconidae, the talon of D-II is usually as large as or larger than in D-III, but in passerines, the claw of D-II is always smaller than D-III (Table 1). Passerines also have significantly less curved claws than do Falconidae, which possess the overall greatest claw inner curvature of raptors (D-I *t*_5_ = 2.015, *p* = 0.050; D-II *t*_5_ = 3.409, *p* = 0.010; D-III *t*_7_ = 1.369, *p* = 0.107; D-IV *t*_7_ = 2.702, *p* = 0.015; Figure 4B). Falconidae have relatively narrow toes compared to other raptors.

### Strigiformes (Owls)

Strigiformes (Figure 1D) are characterised by near uniform large talons on each digit (Table 1), and shorter, robust toes relative to all other raptors, especially on digits 3 and 4 (Claw-I/Toe-I *t*_29_ = 1.731, *p* = 0.047; Claw-II/Toe-II *t*_29_ = −1.241, *p* = 0.112; Claw-III/Toe-III *t*_29_ = 4.976, *p* = 0.00001; Claw-IV/Toe-IV *t*_11_ = 3.627, *p* = 0.002). Strigiformes generally have a lower inner claw curvature than other raptors, with the difference highly significant (99.8%) for D-II (Strigiformes mean = 92.256 degrees, σ = 17.220; Accipitridae + Falconidae + Pandionidae mean = 112.823, σ = 15.964; *t*_29_ = −3.185, *p* = 0.002). Strigiformes can rotate D-IV so that D-II and D-III oppose D-I and the reversed D-IV (respectively): a functionally zygodactyl foot.

### Pandionidae (The Osprey)

Pandionidae (Figure 1E) are characterised by talons that are of nearly uniform large size (Table 1); each digit has strong outer and inner curvature (outer mean = 166.0, σ = 6.405; inner mean = 155.9, σ = 9.067). D-IV bears the largest talon in Pandionidae and can rotate laterally so that it projects posteriorly (functionally zygodactyl) instead of antero-laterally.

Given the time taken to precisely measure each claw (approximately 20 mins), and the lack of availability of feet with splayed toes, it is not feasible to exhaustively measure each of the world's raptor species. However, examination of 223 specimens and 177 photographs of raptors (representing 59 different species; Supporting Information Table S3) failed to find a single specimen that does not conform to the family-level trends we describe here (Accipitridae: hypertrophied D-I and D-II talons; Falconidae: subequally sized talons on each digit, elongate D-III toe; Strigiformes: near uniform large-sized, but weakly curved talons on each digit, short toes; Pandionidae: near uniform large-sized, strongly curved talons on each digit). Ontogeny, gender, and whether the foot was left or right, did not affect the observations described. From this we conclude that our observations apply to all raptor taxa, and that it is most parsimonious that this is related to variation in predatory behaviour among families.

During the preparation of this manuscript, in the character matrix for the phylogenetic analysis of Livezey and Zusi [29], [30], a hypertrophied D-II talon was coded (without further comment) as present for *Accipiter* but absent for *Falco*, *Pandion*, *Strix*, *Otus*, and *Gyps* (all species unspecified). This further confirms the observations we describe herein.

**Figure 5:**
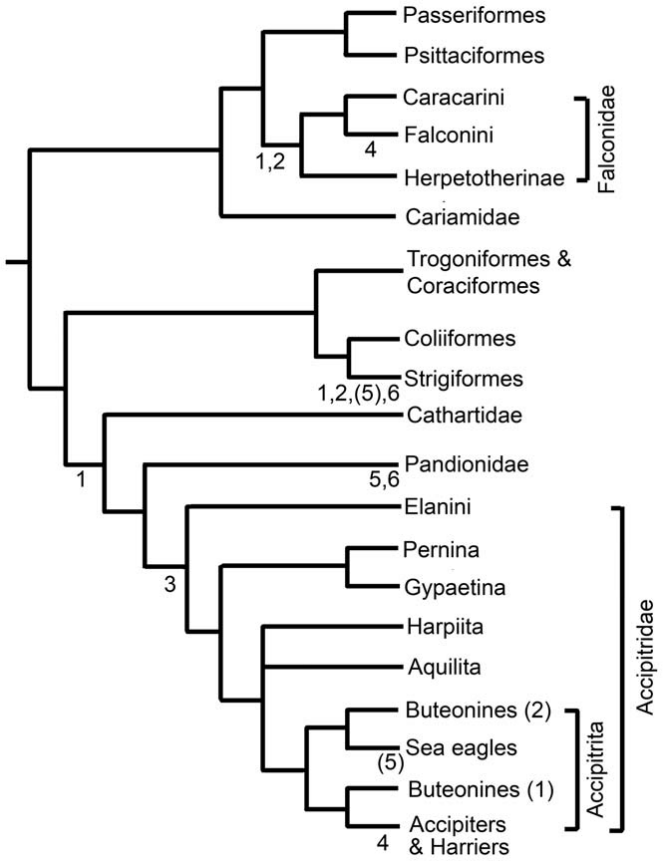
Phylogenetic diagram plotting occurrence of morphologic traits. Numbered traits in parentheses are present only in selected taxa within the clade (see main text). 1. D-II talon as large or larger than D-III; 2. short tarsometatarsus; 3. hypertrophied D-I and D-II talons; 4. elongate toes; 5. highly recurved talons on all digits; 6. subequally large talons on each digit. General arrangement of families after Hackett et al [28]. Nomenclature and arrangement of Falconidae and Accipitridae after Griffiths et al [31], [32] respectively (see main text for exceptions).

In order to facilitate interpretation of traits in a phylogenetic context, the observed family-level trends were plotted onto a cladogram of bird relationships (Figure 5). Literature used in construction of the cladogram include the most recent molecular phylogenetic analyses for Falconidae [31], Accipitridae [32], and birds as a whole [28].

## Discussion

When arranged on a cladogram (Figure 5), the various morphologic trends identified here show expected alignment with family-level clades. Some traits represent unusual departures for a given group, and these can be linked to adaptations related to atypical predatory behaviours (elongate toes in Falconini and *Accipiter*; highly recurved talons on each digit in Pandionidae and fishing eagles; elaborated upon later in discussion). The possession of a talon on D-II that is as large or larger than D-III (which separates raptor from non-raptor taxa) is demonstrated as being independently evolved in Falconidae, Strigiformes, Pandionidae, and Accipitridae, and is presumably related to the predatory behaviour in these clades. The short metatarsus of Falconidae and Strigiformes is also a possible case of convergence, although it is also possible that the elongate tarsometatarsus of Accipitridae is the derived condition.

The observed variation in talon size distribution could simply be the result of phylogenetic inertia: i.e., the tendency for related species to have similar traits because of inheritance from the common ancestral population, rather than it being of adaptive significance. However, given that other variable elements of the foot perform clear functional roles [16], [17], [19], and that there is correlation between claw morphology and function in terrestrial through arboreal birds [25], [34], it is likely that raptor talon morphology does indeed vary dependent on function, since raptor feet are so important for prey capture and manipulation.

We first considered whether talon morphology was primarily affected by diet, but the strong overlap in typical prey choice among raptor families (see Supporting Information Text S1) which exhibit disparate talon and foot morphologies led us to conclude that talon morphology is generally not indicative of diet. One exception may be found in that Falconini and the accipitrid genus *Accipiter* are both avivores, and both have independently evolved elongate/narrow toes (Figure 5). This was also noted by Einoder and Richardson [20]. We also found exception in piscivorous taxa, where all four talons are used to impale fish. In piscivorous taxa, claws are subequally sized, characteristically large, and highly curved. This is seen in the osprey (*Pandion haliaetus*, Figure 1E), the bald eagle (*Haliaeetus leucocephalus*, which possesses talons on D-III and IV that are larger and more curved than expected for Accipitridae), and possibly at least one species of fishing owl (*Ketupa zeylonensis*: the brown fish owl [35]). The convergence of talon morphology among unrelated piscivorous taxa supports our hypothesis that talon morphology corresponds to feeding behavior, and is not merely a phylogenetic artifact.

Variation in hunting technique indirectly affects talon morphology. Falconini strike prey at high velocity, the impact of which may immobilise or seriously impair the victim. The prey of accipitrids and Strigiformes are taken by ambush attacks on or near the ground; as such, they are less likely to be seriously wounded or dead upon capture, being able to struggle against their captor more vigorously. Compared to falconids, accipitrids and Strigiformes must therefore have enhanced ability to restrain struggling prey, and this is partly accounted for by variation in talon and foot morphology. However, in order to fully understand this, variation in prey size must first be considered.

We found that prey restraint and immobilisation strategy changed as prey increased in size, which has only been briefly considered prior to this study [12], [36]. This necessarily occurs across the hypothetical boundary between those prey that can be constricted, and those that are too large to fit within the foot, hence an alternative strategy must be sought. For the purposes of clarity, here we define “small” and “large” to be relative terms that depend on the bodysize of both raptor and prey. “Small” prey are those small enough to be contained entirely within the foot of the raptor (typically encircled by D-III [21]). “Large” prey are those which cannot be contained entirely within the foot. Thus the same prey item might be considered “large” for a small raptor species (e.g., a kestrel), but “small” for a large raptor species (e.g., a peregrine falcon). An intermediate size category does not exist since the terms as defined are discrete not continuous.

Experiments offering laboratory mice to caged raptors [6], [10]–[14] elicited predatory behaviour which we consider specific only to small prey (confirmed by our video observations and also observed for small bird and reptile prey [9]). Here, both immobilisation (constriction) and prevention of escape (containment within the foot) are conducted by the feet, and assisted by beak attacks. Talons are employed only as an aid to prevent escape. This general behaviour is consistently observed in all raptors, with some variation. Falconini have evolved “tomial” or “false teeth” on the beak to help immobilise prey more quickly by severing the spinal cord, or crushing the head [8], [9], [16]. Strigiformes are also known to occasionally perform a twist at the base of the prey's neck, probably also attempting to break it [37]. Accipitrids lack “tomial teeth” and have a weaker bite-force than falconids [17]; they therefore have greater reliance on constriction to immobilise small prey.

For small prey, methods of restraint and immobilisation do not appear to have significant influence on talon morphology in accipitrids and falconids, but are important in Strigiformes, which feed mainly on small prey. Strigiformes have specialised towards maximizing grip strength (increasing their constriction ability) to a greater extent than seen in other raptors [19], which accounts for their unusually enlarged and weakly curved talons. Our measurements show that while overall digit length (including the talon) is similar between Strigiformes and accipitrids of comparable bodysize, Strigiformes' greater claw size (especially of D-III and IV) means that the talon contributes a higher proportion of the overall digit length than in other raptors. Given that the flexor tendons attach to the tubercle at the proximal end of the ungual, short toes combined with extra talon length in owls effectively reduces the lever length of the flexor, thus increasing grip force production, but maintaining the reach of the digit, and approximately the same size enclosable fist. An overall lower curvature for owl talons supports the hypothesis that their increased size is to maintain the reach of the toe. Grip ability and strength is further exacerbated in Strigiformes by the short tarsometatarsus, presence of sesamoids [19], specialized tendon locking mechanism [38] and the zygodactyl foot (which may account partially for Strigiformes' more uniform talon-size distribution: giving a more even grip [20]). Specialising in constriction, Strigiformes rarely take large prey, this being seen more commonly in falconids, and especially accipitrids.

Our analysis found that variation in talon morphology between falconids and accipitrids is most strongly correlated with the change in restraint and immobilisation strategy evident as prey increase in size: recorded here in detail for the first time. When prey are too large to be completely enclosed within the foot, constriction is no longer a viable immobilisation strategy, and because the prey cannot be fully grasped, the raptor must alter its method for preventing escape. We found that for large prey, escape is prevented by the raptor standing atop its victim, using its body weight to pin it down, while holding on with its talons. During the initial energetic struggles that occur immediately after capture of large prey, the additional grip provided by the hypertrophied talons of D-I and II of accipitrids is vital as the raptor tumbles about while keeping latched into its prey, flapping vigorously, trying to gain the upper hand and pin it to the ground. Accipitrids will often use their enhanced grip to drag prey to a new location, to help prevent its escape or conceal the commotion from other predators. Once the prey is suitably immobilised, the raptor proceeds to remove feathers or fur. Accipitrids tend to pluck the back or belly area and will start feeding while the prey is still alive, so long as it does not protest too vigorously. In this prolonged and bloody scenario, prey eventually succumb to massive blood loss or organ failure, incurred during dismemberment. As the prey of accipitrids are often consumed while still alive, a firm grip is constantly required to maintain immobilisation until the prey is dead, further emphasising the importance of the hypertrophied talons of D-I and II. By contrast, Falconini will quickly pluck the neck area and attempt to kill prey swiftly by breaking the neck with a bite attack using the tomial teeth, reducing the necessity for large talons. Falconids also have stronger feet (tarsometatarsus+foot) than accipitrids (which have quicker, more agile feet [17], [18]; see Supporting Information Text S1), and their prey is more likely to be partially injured already from their different hunting strategy. The greater ability of accipitrids to subdue large struggling prey likely accounts for their generally higher rate of predation on large prey than falconids.

Average prey size (ie. the “typical” prey taken by a given raptor species) may represent an important selective pressure driving the morphological evolution of raptor feet. The tendency for Strigiformes to take only relatively small prey has influenced the evolution of their specialized feet. The persistence of a more cosmopolitan, or generalist approach with regards to prey size, has meant that accipitrids and falconids require a more adaptable foot morphology, one that can immobilise both large and small prey, or require adaptations outside of the foot to compensate for possible shortcomings (e.g. the tomial tooth of Falconidae). However, all raptors (potentially excluding specialist piscivores) possess the ability to immobilise small prey, and so it would appear that immobilisation of large prey might represent the strongest selection pressure for mixed-prey-size predators, even if large prey constitute a smaller proportion of the diet (which is subject to many other factors, see Supporting Information Text S1).

Talons are used in manipulating prey during feeding. Unlike owls, which usually swallow prey whole, falconids and accipitrids dismember prey before or during consumption [9]. As observed in the videos, prey (especially small prey) are typically pinned down between the feet by the claws of both left and right D-II, while D-I, III and IV contact the ground, steadying the bird for feeding. To feed, the raptor reaches down between its feet, grabbing tissue in the hooked beak, then pulls upwards, plucking away the feathers or tearing off strips of flesh. We observed two videos in which accipitrines used the enlarged talon of D-II to prise open the body cavity of prey, giving access to the nutritious internal organs.

Additional discussion can be found in Supporting Information Text S1.

### Conclusion

In volant birds, the hindlimb is freed from a primarily cursorial role, allowing specialisation towards perching or foraging. Within all birds of prey, physical attributes of the feet trade off against each other to attain great strength, but it is the variable means by which this is achieved that distinguishes them ecologically. Consequently each taxon has a typical prey and predatory strategy to which it is primarily adapted, but operates within a broad envelope of possible behaviours that may overlap significantly with other taxa.

Our findings show that interdigital talon morphology varies consistently among raptor families, and that this is correlative with variation in their typical prey capture and restraint strategy. We further suggest that change in prey size necessarily causes change in restraint and immobilisation strategy, and that this is the primary factor influencing claw morphology.

This study has important implications for claw functional morphology of fossil bird and non-avian dinosaur taxa that possess proportionally similar claws as those described here. This is currently under investigation by the authors (Fowler et al, in prep).

## Supporting Information

Table S1 Table of all measurements taken, ratios calculated from measurements and used for statistical analysis and comparison.
(0.24 MB XLS)

Table S2 List of behaviour videos, categorised observations and general summaries of important behaviours observed.
(0.06 MB XLS)

Table S3 List of observed taxa, arranged phylogenetically.
(0.03 MB XLS)

Text S1 Additional background information, results and discussion.
(0.06 MB DOC)
