# Supplementary material for: Predatory Functional Morphology in Raptors: Interdigital Variation in Talon Size Is Related to Prey Restraint and Immobilisation Technique
Source: PLoS One. 2009 Nov 25;4(11):e7999. doi: 10.1371/journal.pone.0007999 (PMC2776979; doi:10.1371/journal.pone.0007999)
Supplement: Text S1 — Additional background information, results and discussion. (0.06 MB DOC) [file pone.0007999.s004.doc]

# Supporting Information: Text S1

**Review**

In order to assess the life habits of the extinct taxon *Archaeopteryx*, Peters and Görgner [1] measured an average of inner and outer curvature for each claw, and compared the results to extant birds of known ecologies. Similar studies using only D-III data measured inner curvature [2] or outer curvature [3] to compare *Archaeopteryx* with extant birds that were placed into perceived ecological categories (e.g., perching, trunk climbing, predatory). Glen and Bennett [4] criticised this rigid ecological categorisation, suggesting instead a series of more intermediate ecological groups, and then similarly compared extinct to extant taxa, also based on D-III outer curvature. Csermely and Rossi [5] attempted to differentiate between the talons of raptors and the claws of non-raptorial birds, using a variety of measurements from the toe and claw of D-I and III (considering these the most important digits for either grasping prey or perching). They found that a combination of size and curvature segregated Strigiformes from Falconiformes (Accipitridae + Falconidae) and non-raptors, but they could not fully distinguish Falconiformes from non-raptors. In the study most relevant to our own, Einoder and Richardson [6] took foot measurements from a range of extant Australasian raptors, looking for ecological links with prey choice, size, “hunting-killing technique”, and phylogeny. Although Einoder and Richardson [6] take many measurements in their study, they did not assess interdigital talon size directly nor did they measure claw curvature. Instead they concentrated on toe-to-talon ratios, which are susceptible to unusually large or small claws / long or short toes affecting the result. Moreover, since they measure claw length as a straight line from the claw tip to the beginning of the talon cuticle, highly curved claws would appear smaller than the same sized claw that was not highly curved. It is not clear how much this affects their findings. While some findings from our study were also noted by Einoder and Richardson (e.g., an enlarged D-II talon in Accipitridae), most were not (e.g., large-sized, low-curvature claws in Strigiformes; high curvature in piscivores), presumably since many of the trends we identify are not as easily recognised using toe-to-talon ratios alone.

**Background information**

Various physical attributes strongly influence the effectiveness of the hindlimb as a predatory device. The development of a large flexor tubercle at the proximal end of the ventral side of the talon can give a good indication of strength of the foot, for it is the distal attachment point for the flexors *digitorum longus* and *hallucis longus*, the primary muscles for clenching the feet [7]. Shortening and broadening the tarsometatarsus, as seen in Strigiformes and Falconidae, decreases the lever length and increases accommodation for these muscles, increasing grip strength at the cost of stride length, or “quickness” of the feet [8]. Conversely, the longer tarsometatarsus of accipitrids gives them quicker feet than other raptors, but at the cost of lesser strength [9]. In grasping animals, the digits, especially the distal phalanges, are elongated, whereas the opposite is true of cursorial taxa [10]. However, the longer a digit becomes, the greater the length of the out-lever, so that grip strength decreases; consequently, graspers that require a strong grip tend to have elongate distal phalanges but foreshortened proximal phalanges [10].

Prey choice, even within a single species, is variable and depends on factors such as geographical location, seasonality, abundance, ease of capture, and the particular hunting preferences of the individual bird [e.g., 11, 12]. Nevertheless, general statements can be made about food preferences of sampled taxa. Falconini and species of *Accipiter* (Accipitridae: Accipitrinae) are characterized mainly as avivores [13], although insects or mammals can be important to some taxa [11, 14]. Buteonines (Accipitridae) are observed to take carrion more commonly than falconini do, although mammals taken live comprise most of their diet; golden eagles are even known to take small ungulates [15, 16]. The bald eagle, however, is mostly piscivorous, as is the osprey (Pandionidae). Strigiformes feed mainly on small mammals.

The main hunting strategies employed by raptors can be placed into five categories: stoop; level-attack; harrying; watch and strike; and ground attack. In a stoop the raptor makes a controlled dive onto prey either mid-air, or on or near the ground. During a stoop, the raptor typically clenches D-II to IV into a fist upon impact, with D-I left extended to strike through the prey [17]. Larger prey may be killed outright by a strike, due to higher inertia [18]. In a level attack the prey is struck in mid-air from level flight; harrying occurs when prey are constantly harried at low level; watch and strike is when the raptor surveys prey from a perch, soar, or hover before flying a short distance to attack it; and ground attack is where the prey is pursued and captured on foot.

Although different raptors are physically specialised for a particular hunting technique, they can employ alternate methods depending on the conditions and expected escape strategy of the prey [19]. However, the further a predatory attack technique deviates from that for which the predator is best adapted physically, the less successful it is likely to be [20, 21].

Falconini are highly maneuverable in flight and this forms the basis of their typical hunting strategy, striking prey in mid-air by either a stoop or level-flight attack. Falconini do not always keep hold of their prey after the initial strike [14, 17], but may come around for another attack, or if the prey is already mortally wounded, follow it to the ground. Accipitrids are very rarely successful in mid-air attacks, where the flight of the prey item is unrestricted [20]. Instead, along with Strigiformes, accipitrids normally ambush prey while it is on or near the ground, usually spotting them from a perch or high soar.

**Methodology**

We followed the methodology of Pike and Maitland [3] for our measurements (Figure 2). We improved upon their method by taking measurements using Adobe Photoshop^TM^ rather than printing photos and taking measurements manually. Glen and Bennett [4] criticised the method of Pike and Maitland [3], citing it as less accurate than their own manual method using a specially designed protractor. Both methods were tested by DWF, EAF, and JBS for a small sample of claws. We found more consistent results using our Adobe Photoshop^TM^ method (average standard deviations 0.71 degrees and 0.10 mm), rather than the protractor method of Glen and Bennett [4] (average standard deviations 5.83 degrees and 0.46 mm).

Arc Length (AL, see figure 2) was calculated using basic circle geometry and trigonometry utilizing the measurements A: the straight line (chord) distance from claw base to tip, and O: the angle of curvature. Since A is bisected by the radius of the circle (part of the radius is represented by the measurement Hm: height of claw at midpoint) then this forms a right-angled triangle of which ½ x A is the opposite side to the angle (which is also bisected, and hence is ½ x O), and a complete radius is the hypotenuse. We then used a trigonometric equation to calculate the radius:

opposite = hypotenuse x sin (angle)

therefore ½A = radius x sin ½O

therefore ½A / (sin ½O) = radius

Once the radius (r) is known, AL can be calculated as a proportion of the circumference of a circle (2πr), where:

Proportion of circumference (AL) = circumference x percentage of circle

Therefore AL = 2πr x O/360

Therefore AL = 2π x (½A / (sin ½O)) x O/360

The same formulae can be used for calculating either ALo (outer) or Ali (inner), so long as the corresponding outer and inner values for A and O are used.

Figure 2 confirms that our modeled curvature matches the observed curvature of bird claws as circular [3] rather than a logarithmic spiral, which is seen in certain mammals [22].

The absolute size of claws is straightforward to observe in specimens, but difficult to compare quantifiably among specimens because of their claws compared to their toe lengths or tarsometatarsus length. For example, if a bird has a larger claw/toe ratio than another, this could be due either to a large claw or a short toe. Division of claw arc length by body mass is one option, but body mass data are not widely available for specimens, and may be an inaccurate representation of a healthy body mass in life, if the data were taken immediately before or after death. To solve this problem, measurements were converted into ratios relative to digits III or IV, which have less size variability than digits I or II in most taxa, and thus standardize the measurements effectively for comparisons.

Behavioural data was taken from over 170 videos of predatory activity (Supporting Information Table S2). A minimum of 48 species of raptor (approximately 150 individual birds) are featured in the videos (some taxa are only identifiable to genus level). Videos were collected from websites across the internet, comprising a mixture taken by professionals and the public. Videos are available from the authors on request.

Photographs of some of the sampled specimens can be found on Morphobank:

http://morphobank.geongrid.org/

**Vetted data set**

Four specimens (*Harpia* AMNH-1383, *Aquila* AMNH-GE-50, *Cariama* AMNH-1392, and *Sagittarius* AMNH-4253) lacked the keratin sheath on one or more claws. The full sets of measurements were collected for these specimens, but excluded from the correspondence analysis in Figure 3. When these specimens are included in the analysis, they tend to plot near related taxa, indicating that if valid keratin sheath measurements were obtained, these additional data points would support the trends currently observed.

When included in the correspondence analysis, the members of the growth series of *Bubo* *virginianus* cluster tightly together. For the final analysis, however, the juveniles and subadults were excluded to prevent any noise from ontogenetic variation.

Including relative tarsometatarsus length (length of tarsometatarsus divided by total length of D-III including claw) introduced noise to the data set, disrupting the clear clustering of families observed when only considering toe lengths and claw sizes. Because *Sagittarius* and *Cariama* have unusually long metatarsi relative to toe length, including them with a metatarsal analysis yielded such strong outliers that relationships amongst other taxa were clouded. Thus, tarsometatarsus length was not a factor in the final analysis (Figure 3).

Several specimens (*Halliaeetus* MOR ECO-4685, *Circus* MSU ECO-4732, *Spizella* MSU ECO-“tree sparrow”, and *Icterus* MSU ECO-teaching B. oriole) had well preserved left and right feet. All other specimens only had fully preserved and accessible claws on one foot. For those specimens with both feet preserved, all measurements for left and right digits were averaged, and these averaged values used for analysis with the rest of the data set. The mean differences in measurements between left and right feet of the same individuals ranged between 0.9 and 2.4 percent, and were only significantly different in the specimen of *Circus* (MSU ECO-4732, *p*<0.05); despite this, both feet of MSU ECO-4732 demonstrated the expected claw size distribution for Accipitridae.

Because most specimens were measured from a single foot, and it is unknown whether that foot was slightly larger or slightly smaller than its counterpart, there could be noise introduced into the data set that would have been prevented by only including averages of left and right feet from each individual. However, this would severely limit the size of the data set. Because the percentage difference in claw size between left and right feet is usually insignificant, we are confident that using measurements from a single foot or an average of both feet produces a reliable data set.

**Results**

For measurements and ratios see Supporting Information Table S1. For video behavioural data see Supporting Information Table S2.

Institutional abbreviations: (**MOR-OST**) Museum of the Rockies osteology collection, Bozeman, Montana; (**MSU-ECO**) Montana State University, Dept. of Ecology, Bozeman, Montana; (**MSU-ESCI**) Montana State University, Dept. of Earth Sciences, Bozeman, Montana; (**AMNH**) American Museum of Natural History, New York City.

**Discussion**

Our measurements confirm that in all sampled raptors and most other birds, claws taper evenly toward the tip. There are some exceptions, notably woodpeckers (Picidae) and similar trunk-climbing birds, in which tapering is weak until near the tip, whereupon it becomes finely constricted in a logarithmic spiral [1, 3]. Our observations confirm that in most birds (including Accipitridae and Falconidae, *contra* Peters and Görgner [1]), claw cross-section is arched [23], with the exception of the osprey and certain Strigiformes (usually only D-I) where the cross-section is tubular with a rounded ventral keel.

Although all accipitrids possess hypertrophied talons on D-I and D-II, some photographic evidence suggests that basal members of the clade (notably, the Elanini) possess smaller D-I and D-II talons than was observed for more derived taxa (e.g. *Accipiter*, *Buteo*, *Aqulia*). Without a specimen of *Elanus* from which to take precise measurements, it is difficult to confirm this, but it would be corroborative with the hypothesis that the most derived clades of Accipitridae possess particularly hypertrophied D-I and D-II talons.

Most Accipitridae possessed a single row of enlarged keratinous scales on the anterior side of the tarsometatarsus, exaggerated into a single elongate scale in the sharp-shinned hawk, *Accipiter striatus*. The tarsometatarsus of the golden eagle (*Aquila chrysaetos*) is feathered. In the bald eagle (*Haliaeetus leucocephalus*), Falconidae and Pandionidae there are two or more rows of smaller scales present. The tarsometatarsus of Strigiformes is feathered. It is possible that these large scales and/or feathers help to shield the metatarsi of accipitrids and Strigiformes from retaliatory attacks by their more able prey. Falconidae, Pandionidae, and the bald eagle might not require such elaboration as their prey are typically more subdued upon capture, or as in the case of fish, lack the physical capability to retaliate effectively.

Within non-raptors the D-II claw was relatively larger than expected in the common crow, *Corvus*, (D11/DIII *z*=1.650, *p*=0.049) and turkey vulture, *Cathartes aura* (D11/DIII *z*=2.385, *p*=0.009). The slightly enlarged D-II claw in both these taxa is probably an adaptation toward carcass processing. As in raptors dismembering prey, the D-II claw of
many scavengers is often used to pin down the food item while the beak tugs off bits of flesh.

The suggestion of Csermeley and Rossi [5] that the talons of D-I and III are the most important for perching or grasping and holding prey, does not seem consistent with our observations that in all birds sampled the D-III talon typically exhibits the least curvature, and that the talons on D-I and II, not D-I and III, are hypertrophied in Accipitridae (and to a much lesser extent in Falconidae). Their assertion is correct in that D-III, the longest digit, is the most important for wrapping around a prey item or perch, but D-I and II, the shortest digits, can probably exert the most leverage; hence it is logical that these should bear the largest talons. D-I and II are also positioned on the inside of each foot, which facilitates holding and manipulating prey when using both feet. This reinforces the hypothesis that the foot functions as a whole, but each digit plays an important separate part.

Mantling (the raptor wrapping its wings around the victim) was a common observation, especially while the prey was still alive, but seemed less common once the prey was dead or immobilised. Mantling behaviour is often thought to be an attempt to mask prey from other predators, lest they attempt piracy [e.g., 14]. It is possible that increased mantling of live prey is an attempt to hide the commotion of a victim struggling against immobilisation. Once the prey is immobilised, mantling becomes less necessary. It is also plausible that mantling also helps limit the escape of live prey.

Hunting involves great physical exertion; raptors are often observed panting heavily in the immediate aftermath of an attack, and in order to recover, might not begin immobilisation for a minute or so. In this case the raptor must successfully keep hold of its struggling prey before immobilisation commences.

**References**

1 Peters DS, Görgner E (1992) A comparative study on the claws of *Archaeopteryx*. In: Campbell KE, editor. Papers in avian paleontology. Honoring Pierce Brodkorb. Los Angeles: Science Series Natural History, Museum of Los Angeles County. pp. 29-37.

2 Feduccia A (1993) Evidence from claw geometry indicating arboreal habits of *Archaeopteryx*. Science 259: 790–793. ([doi:10.1126/science.259.5096.790](http://dx.doi.org/10.1126/science.259.5096.790))

3 Pike AVL, Maitland DP (2004) Scaling of bird claws. J. Zool. 262: 73-81. ([doi:10.1017/S0952836903004382](http://dx.doi.org/10.1017/S0952836903004382))

4 Glen CL, Bennett MB (2007) Foraging modes of Mesozoic birds and non-avian theropods. Curr. Biol*.* 17: R911-R912.

5 Csermely D, Rossi O (2006) Bird claws and bird of prey talons: Where is the difference? Ital. J. Zool. 73: 43–53.

6 Einoder L, Richardson A (2007) Aspects of the hindlimb morphology of some Australasian birds of prey: a comparative and quantitative study. Auk 124: 773-788.

7 Goslow GE (1972) Adaptive mechanisms of the raptor pelvic limb. Auk 89: 47–64.

8 Ward AB, Weigl PD, Conroy RM (2002) Functional morphology of raptor hindlimbs: implications for resource partitioning. Auk 119: 1052–1063.

9 Sustaita, D. 2008. Musculoskeletal Underpinnings to Differences in Killing Behavior Between North American Accipiters (Falconiformes: Accipitridae) and Falcons (Falconidae). - J. Morphol. 269: 283–301. (doi: 10.1002/jmor.10577)

10 Hopson JA (2001) Ecomorphology of avian and nonavian theropod phalangeal proportions: Implications for arboreal versus terrestrial origins of bird flight. In: Gauthier JA, Gall LF, editors. New Perspectives on the Origin and Early Evolution of Birds: Proceedings of the International Conference in Honor of John H. Ostrom. New Haven: Peabody Museum of Natural History. pp. 210-235.

11 Reynolds RT, Meslow EC (1984) Partitioning of food and niche characteristics of coexisting Accipiters during breeding. Auk 101: 761-779.

12 Lewis SB, Titus K, Fuller MR (2006) Northern Goshawk Diet During the Nesting Season in Southeast Alaska. J. Wildlife Manage. 70: 1151–1160. ([doi:10.2193/0022-541X(2006)70[1151:NGDDTN]2.0.CO;2](http://dx.doi.org/10.2193/0022-541X(2006)70%5B1151%3ANGDDTN%5D2.0.CO%3B2))

13 Hertel F (1995) Ecomorphological indicators of feeding behavior in recent and fossil raptors. Auk 112, 890-903.

14 Johnsgard PA (1990) Hawks, eagles and falcons of North America. Washington, DC : Smithsonian Institution Press. 403 p.

15 Brown LH, Watson A (1964) The Golden Eagle in relation to its food supply. Ibis 106: 78-100.

16 Matchett MR, O'Gara BW (1987) Methods of controlling golden eagle depredation on domestic sheep in southwestern Montana. J. Raptor res. 21: 85-94.

17 Goslow GE (1971) The attack and strike of some North American raptors. Auk 88: 815–827.

18 Bond RM (1936) Eating habits of falcons with special reference to pellet analysis. Condor 38: 72-76. ([doi:10.2307/1363552](http://dx.doi.org/10.2307/1363552))

19 Watson, J. 1997. The Golden Eagle. London: T. and A.D. Poyser. 392 p.

20 Rudebeck G (1950) The choice of prey and modes of hunting of predatory birds with special reference to their selective effect. Oikos 2: 65-88.

21 Rudebeck G (1951) The choice of prey and modes of hunting of predatory birds with special reference to their selective effect (cont.). Oikos 3: 200-231.

22 Mattheck C, Reuss S (1991) The claw of the tiger: An assessment of its mechanical shape optimization. J. theor. Biol. 150: 323-328. ([doi:10.1016/S0022-5193(05)80431-X](http://dx.doi.org/10.1016/S0022-5193(05)80431-X))

23 Manning PL, Payne D, Pennicott J, Barrett PM, Ennos, RA (2006) Dinosaur killer claws or climbing crampons? Biol. Lett. 22**:** 110-112. ([doi:10.1098/rsbl.2005.0395](http://dx.doi.org/10.1098/rsbl.2005.0395))
